# Sex Disparities of Genomic Determinants in Response to Immune Checkpoint Inhibitors in Melanoma

**DOI:** 10.3389/fimmu.2021.721409

**Published:** 2021-11-02

**Authors:** Fuyan Shi, Wenjing Zhang, Yichen Yang, Yitao Yang, Junyi Zhao, Mengqi Xie, Chao Sheng, Suzhen Wang, Qinghua Wang

**Affiliations:** ^1^ Department of Health Statistics, Key Laboratory of Medicine and Health of Shandong Province, School of Public Health, Weifang Medical University, Weifang, China; ^2^ Tianjin Cancer Institute, National Clinical Research Center for Cancer, Key Laboratory of Cancer Prevention and Therapy of Tianjin, Tianjin Medical University Cancer Institute and Hospital, Tianjin, China; ^3^ Department of Epidemiology and Biostatistics, National Clinical Research Center for Cancer, Key Laboratory of Molecular Cancer Epidemiology of Tianjin, Tianjin Medical University Cancer Institute and Hospital, Tianjin, China

**Keywords:** sex disparities, biomarkers, immunotherapy, SMGs, mutational signatures, molecular subtypes, melanoma

## Abstract

**Background:**

Despite the acknowledged sex-related differences in immune response and immune checkpoint inhibitor (ICI) efficacy, little is known about the sex disparities in melanoma of novel genomic determinants for ICI therapies.

**Methods:**

Pretreatment genomic profiles and clinical characteristics of 631 melanoma patients treated with ICIs (i.e., inhibitors of CTLA-4, PD-1/PD-L1, or both) were comprehensively curated. Genomic factors, i.e., significantly mutated genes (SMGs), mutational signatures, and molecular subtypes were identified, and their associations with ICI treatment efficacy in male and female patients were evaluated.

**Results:**

Of the 15 SMGs identified in this study, three genes (i.e., *CFH*, *DGKG*, and *PPP6C*) were found to exhibit sex differences with respect to ICI efficacy. Among these, *CFH* mutations exhibited both response rate and survival benefits in male, but not in female patients. A total of four mutational signatures (i.e., signatures 1, 4, 7, and 11) were extracted. Male patients with signature 4 (also known as smoking-related signature) had an inferior ICI response rate and overall survival. However, this association was not significant in females. An immune subtype based on mutational activities was found to be significantly associated with poor ICI survival in female patients.

**Conclusion:**

We uncovered several sex-dependent genomic correlates of response to ICI treatment, such as male-biased *CFH* mutations and signature 4 and the female-biased immune resistance subtype. The findings derived from this research provide clues for exploring different immunotherapeutic approaches in male and female patients with melanoma.

## Introduction

Sex is an important factor that influences immune system response to multiple antigens (1). On average, females have stronger innate and adaptive immune responses than males (1). It has been reported that substantial sex-based differences in the immune system could be related to the natural course of chronic inflammatory diseases, such as cancer (2, 3). For example, males have an approximately twice the mortality risk than females in melanoma and lung and bladder cancers (2, 3).

Cytotoxic T-lymphocyte protein 4 (CTLA-4) and programmed death receptor-1 (PD-1) signals play vital roles in the immune escape, and they are increasingly being recognized as immunotherapy targets in several advanced cancers (4). Recent animal studies have reported the roles of sex hormones for the expression and function of PD-1/PD-L1 signaling (5–7), and the distinct treatment responses of an anti-PD-L1 agent have been observed in female and male mice in melanoma murine models (8).

Based on the existing studies and results, multiple larger meta-analyses of immune checkpoint inhibitor (ICI) efficacy were performed and demonstrated that male patients could obtain relatively more benefit from ICI therapies than females (9, 10), and this finding is supported by two reasonable and complementary pieces of evidence. The first is relevant to sex dimorphism in the immune response. As described previously, the lower risk of cancer mortality could be exhibited in female patients owing to their stronger immunity than male patients. However, the more efficient immune surveillance mechanisms could make the tumors in advanced females less immunogenic and more easily escaped (11). This process of tumors in female patients results in more resistance to ICI treatments. The second evidence is relevant to the sex differences in cancer biology. Tumor mutation burden (TMB) is recognized as a promising indicator for predicting ICI clinical benefits (12). Several recent studies have revealed that male patients had a markedly higher TMB than female patients (13, 14). Besides, male patients are more likely to undergo exposure to ultraviolet light and tobacco smoking, which could induce an elevated mutational burden.

A series of molecular biomarkers have been identified for the responses to ICI therapies, such as TMB, PD-L1 protein expression (15), T-cell-inflamed gene expression profile (GEP) (16), and cytolytic activity (CYT) (17). Owing to the limitations of these biomarkers in predicting ICI clinical outcomes, substantial efforts have been undertaken to explore novel determinants of ICI efficacy. Mutations in single genes, such as *POLE* (18), *PBRM1* (19), *JAK1*/*JAK2* (20), and *B2M* (20), were associated with preferable or inferior ICI outcomes. Tumors with specific mutational signatures have been reported to correlate with distinct ICI responses. Lung cancer patients with tobacco smoking-related or APOBEC-related signatures exhibited a favorable ICI clinical outcome compared with those without such signatures (20). A positive association between ultraviolet light exposure-related mutational signature and ICI benefits in melanoma has also been reported (20). Immune molecular subtypes have been previously identified and employed to evaluate tumor prognosis and immunotherapy efficacy. Potential immune subtypes can be obtained by clustering the mutational activities extracted from mutational profiles (21). Taking into account sex-based immunological and immunotherapy differences, Ye et al. conducted comprehensive analyses to explore the sex bias of related immunotherapy biomarkers to better understand sex dimorphism in ICI efficacy (10).

Although multiple indicators of ICI efficacy have been demonstrated, as described previously, only a subset of melanoma patients could benefit from this therapy. Therefore, novel and more effective biomarkers are urgently needed, and their associations with ICI responses in distinct sex subgroups needed to be elucidated. In this study, we curated an expanded clinically annotated ICI-treated melanoma cohort to identify novel significantly mutated genes (SMGs), mutational signatures, and immune molecular subtypes specific to male or female patients.

## Methods

### Collection of Genomic Data and Clinical Information

All pretreatment somatic mutations and clinical information of the aggregated melanoma cohort (a total of 631 samples) were curated from previous eight immunotherapy studies (20, 22–28). The somatic mutational profiles were uniformly annotated with the Oncotator (29) against the h19 reference genome. Gene expression profiles were obtained from four of eight included cohorts, namely Hugo et al. (25), Riaz et al. (26), Van Allen et al. (22), and Liu et al. (28) cohorts. Detailed clinicopathologic data including age, sex, stage, ICI response status, follow-up information, and ICI types of the eight curated studies are shown in **Table S1**. Objective response rate (ORR) reflects the fraction of patients with complete response (CR) or partial response (PR); other statuses, including stable disease (SD) and progressive disease (PD), were considered not to be efficacious to ICI treatment.

Somatic mutation data, mRNA expression profiles, and clinical information of 457 melanoma samples from the Cancer Genome Atlas (TCGA) were downloaded from Genome Data Commons (https://gdc.cancer.gov) for the specific comparison.

### Identification of SMGs

SMGs were identified by applying both MutSigCV (30) and OncodriveCLUST (31) algorithms against the hg19 genome. MutSigCV detects the significant enrichment of non-silent somatic alterations in one gene by taking into consideration the background mutation rate estimated through silent mutation. OncodriveCLUST is based on the fact that most of the variants in cancer-causing genes are enriched at few specific loci (i.e., hot spots). This method takes advantage of such positions to identify cancer driver genes. In addition, to be statistically significant identified by the above two algorithms (both *q* < 0.1), a candidate SMG must meet the criterion of expressing in the TCGA melanoma dataset (32). The mutational patterns of SMGs were visualized with R package maftools (33).

### Deciphering Mutational Signatures Operative in the Genome

The algorithm proposed by Kim et al. (34) was used to detect mutational signatures from the aggregated 631 melanoma samples. The core of this method is the Bayesian variant non-negative matrix factorization (NMF), which can automatically determine the optimal rank of mutational signatures. Specifically, NMF was used to decompose mutation portrait matrix ***A*** that contained the 96 base substitution classes with trinucleotide sequence pattern. Matrix ***A*** was factorized into two non-negative matrices ***W*** and ***H*** (i.e., ***A*** ≈ ***WH***), where ***W*** reflects the extracted mutational signatures and ***H*** indicates the mutation activities of each corresponding signature. The column of matrix ***A*** is the count of detected signatures and rows representing the 96 base substitution types, which are the permutation and combination of six main mutational categories (i.e., C > A, C > G, C > T, T > A, T > C, and T > G) and their surrounding adjacent bases. The rows and columns of matrix ***H*** indicate the individual signatures and their corresponding mutational activities, respectively. All extracted mutational signatures were then compared and annotated against the 30 curated signatures stored in the COSMIC (version 2) database based on the cosine similarity.

### Detection of Potential Molecular Subtypes

We used consensus clustering to investigate potential molecular subtypes of the aggregated melanoma cohort. After extracting the activities of existing mutational signatures of all patients, we subsequently used the partition around medoids (PAM) algorithm with the Euclidean distance metric and performed 500 bootstraps each comprised 80% of patients of the integrated cohort. Clustering rank was assessed from 2 to 10, and the optimal rank was identified by evaluating the cluster consensus coefficient. The consensus clustering analysis was performed by using the R package ConsensusClusterPlus (35).

### Estimation of Distinct Immune Cell Infiltration With Single-Sample Gene Set Enrichment Analysis

The single-sample gene set enrichment analysis (ssGSEA) method (36) was used to quantify the infiltration abundance of 28 immune cell subtypes that are overrepresented in the tumor microenvironment. Specific feature gene panels for each immune cell type were collected from a recent study (37) and curated in **Table S2**. The relative infiltration level of each immune cell subset was represented by an enrichment ssGSEA score calculated from the R package GSVA (36).

### Estimation of Tumor Infiltration Lymphocytes

The CIBERSORT algorithm (38) was used to calculate the proportion of infiltration immune cell subsets in tumors, which is a useful approach to provide an estimation of the abundances of 22 human hematopoietic cell phenotypes with 547 genes from the leukocyte gene signature matrix, termed LM22.

### Comprehensive Analyses of Immune Checkpoint Genes

An integrated list of 34 immune checkpoint genes was curated from a recently published immunotherapy research (10). In this study, the expression of *VISTA* was not found in the aggregated transcriptomic profile. We analyzed the distinct distributions of 33 immune checkpoint genes based on distinct subgroups.

### GSEA and Network Analysis

The functional pathway-level changes in samples with different feature subgroups were evaluated. R packages limma (39) and edgeR (40) were employed to perform differential analyses of each gene in distinct subgroups. Especially, read counts of gene expression profile were normalized by calcNormFactors function in the package edgeR, and then as input to lmFit and eBayes functions in the limma package. The differential *t* statistics obtained from eBayes function were then applied to run GSEA implemented by fgsea package (http://bioconductor.org/packages/release/bioc/html/fgsea.html). Cell signaling pathways in Kyoto Encyclopedia of Genes and Genomes (KEGG) were utilized as background datasets. The false discovery rate (FDR) and normalized enrichment score (NES) were calculated based on one million permutations.

### Determination of TMB and NB

In this study, TMB was regarded as the log2 transformation of total non-synonymous mutations per megabase. In the multivariate logistic regression model, TMB was stratified as high and low subgroups with the median. The available neoantigen data of 340 melanoma patients were obtained from The Cancer Immunome Atlas (TCIA, https://www.tcia.at/home). Neoantigen burden (NB) was the total count of neoantigen in each sample and was also log2 transformed.

### Statistical Analyses

All statistical analyses were performed by using R software (version 4.0.2). Kaplan–Meier survival analyses were achieved with R packages survival and survminer, and a log-rank test was used to compare the significant differences between survival curves. Multivariate Cox and logistic regression models implemented by forestmodel package were conducted to adjust the confounding variables, such as age, sex, stage, and ICI treatment types. The association of continuous and categorical variables with distinct subgroups was calculated with Wilcoxon rank-sum test and Fisher exact test, separately. Results were considered to be statistically significant if the two-sided *P*-values were less than 0.05.

## Results

### Sex-Biased Pretreatment Genomic Features and SMGs

A total of 333,968 pretreatment non-synonymous somatic variants were obtained from eight previously published studies with a total of 631 ICI-treated melanoma patients (a median of 257 mutations per sample). Overall, there were 364 (57.7%) male and 199 (31.5%) female patients, and sex information of the remaining patients (10.8%) was unavailable. The other detailed clinical characteristics are listed in **Table S1**. We observed that elevated TMB was markedly associated with favorable ICI overall survival (OS) (HR: 0.91, 95% CI: 0.87–0.96, *P* < 0.001; **Figure S1A**) and higher response rate (HR: 0.84, 95% CI: 0.76–0.92, *P* < 0.001; **Figure S1B**) in multivariate regression models, which was consistent with previously reported findings (12). We then investigated the sex bias of TMB and found that male melanoma patients harbored a significantly higher TMB than female patients (median TMB: 3.27 *vs*. 2.83, Wilcoxon rank-sum test *P* = 0.003; **Figure S2A**). This association was statistically significant even after adjusting for age, stage, and ICI type (HR: 1.48, 95% CI: 1.03–2.15, *P* = 0.036; **Figure S2B**). This finding further supports the evidence that male melanoma patients might benefit more from ICI therapies than female patients.

**Figure 1:**
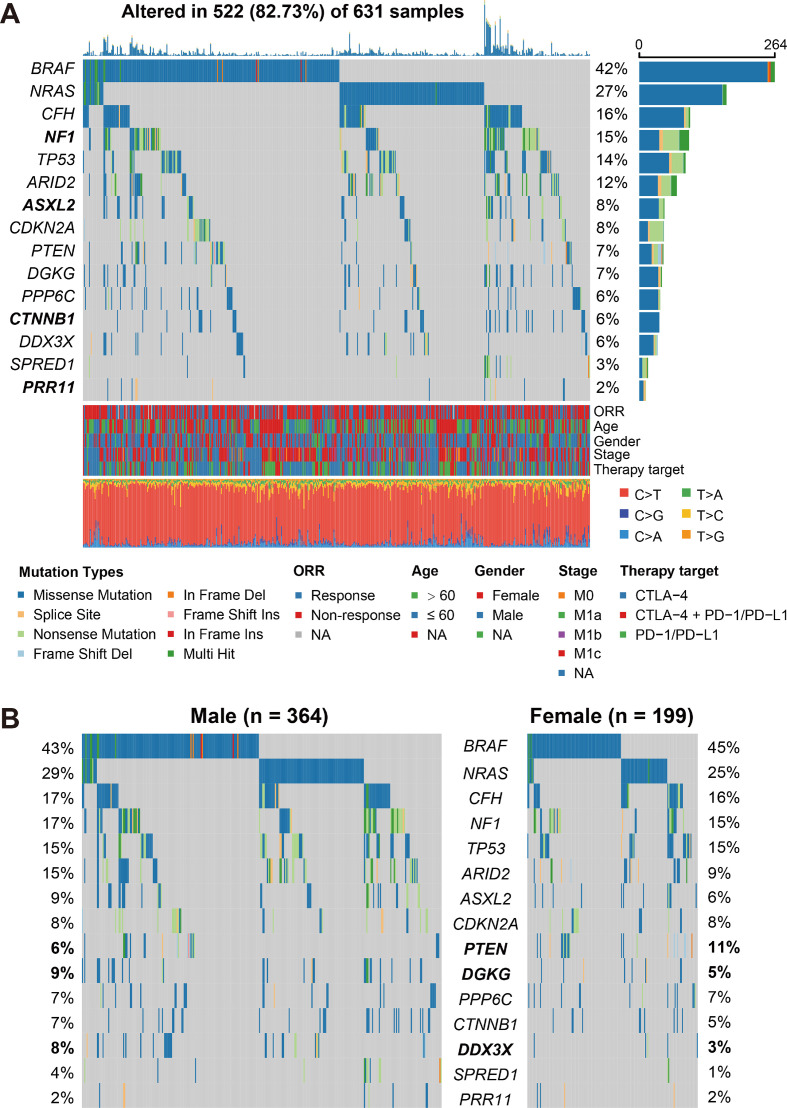
Identification of significantly mutated genes (SMGs) in the aggregated melanoma cohort and their sex-biased mutation distributions. **(A)** Waterfall plot representation of mutational patterns of the identified SMGs. The left panel shows the gene symbols, the upper panel indicates the non-synonymous mutation counts for each patient, the middle plot illustrates the mutational patterns of the identified 15 SMGs with different mutation types colored differently, the right panel shows the mutation rate of each SMG, and the bottom panel indicates the clinical characteristics and base substitution categories. Genes highlighted in bold are the SMGs that were not previously reported. **(B)** Distinct mutation rates of the identified 15 SMGs between male and female patients. SMGs that were significantly differentially mutated between the two subgroups were highlighted in bold.

We employed MutSigCV and OncodriveCLUST algorithms to re-annotate SMGs. In total, 15 SMGs were identified (**Figure 1A**), including the well-known melanoma driver genes (e.g., *BRAF*, *NRAS*, *NF1*, *TP53*, and *PTEN*) and four novel SMGs (i.e., *CFH*, *ASXL2*, *CTNNB1*, and *PRR11*). We subsequently evaluated the distinct mutation rates of all 15 SMGs in male and female subpopulations. Results showed that female patients exhibited a higher mutation rate of *PTEN* (11% *vs*. 6%). However, higher mutation rates of *DGKG* (9% *vs*. 5%) and *DDX3X* (8% *vs*. 3%) were observed in male patients (Fisher exact test, all *P* < 0.05; **Figure 1B**).

### Association of SMG Mutations With ICI Efficacy in Male and Female Patients

**Figure 2:**
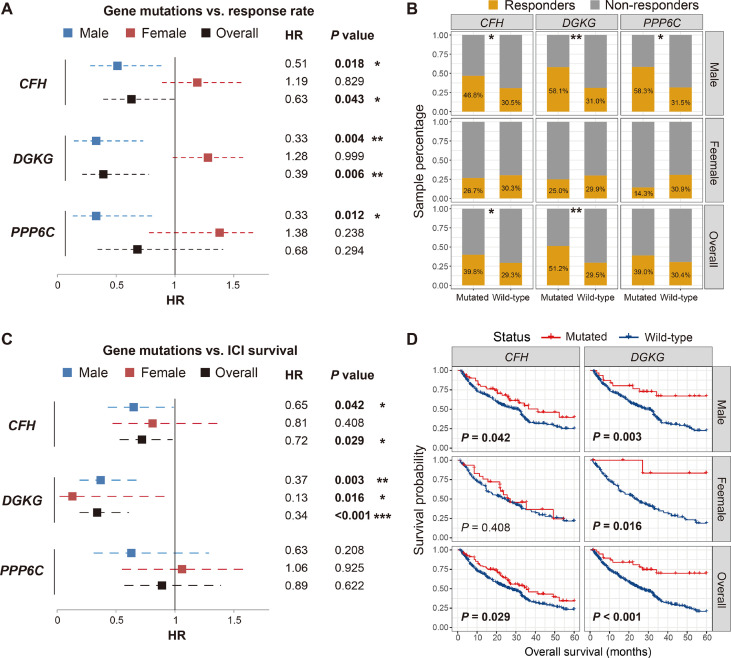
Association of *CFH*, *DGKG*, and *PPP6C* mutations with immune checkpoint inhibitor (ICI) response rate and overall survival benefits in distinct sex groups. **(A, B)** Forest plot and bar plot representations of the association of SMG mutations with ICI response rate in distinct sex groups. **(C, D)** Forest plot and survival plot representations of the association of SMG mutations with ICI survival outcome in distinct sex groups. *P < 0.05; **P < 0.01; ***P < 0.001.

We conducted comprehensive analyses on the association of the 15 identified SMG mutations with ICI efficacy (i.e., response rate and survival outcome) in male, female, and overall melanoma patients. Results of the response rate association demonstrated that of all 15 SMGs, three (i.e., *CFH*, *DGKG*, and *PPP6C*) mutations were identified to exhibit sex-dependent ICI responses (**Figure 2A**). Male patients with *CFH* mutations had a significantly higher ICI response rate than those without such mutations (46.8% *vs*. 30.5%, Fisher exact test *P* < 0.05; **Figure 2B**). A positive association between *CFH* mutations and ICI response was also observed in all patients (39.8% vs. 29.3%, Fisher exact test *P* < 0.05; **Figure 2B**). However, no significant difference was detected in female patients (26.7% *vs*. 30.3%, Fisher exact test *P* > 0.05; **Figure 2B**). *DGKG* mutations were also associated with higher response rates in male (58.1% *vs*. 31.0%, Fisher exact test *P* < 0.01; **Figure 2B**) and overall patients (51.2% *vs*. 29.5%, Fisher exact test *P* < 0.01; **Figure 2B**), but not in female patients. *PPP6C* mutations were identified to associate with an elevated ICI response rate only in male patients (58.3% *vs*. 31.5%, Fisher exact test *P* < 0.05; **Figure 2B**), and no significant associations were found in female and overall patients.

We then evaluated the association of the abovementioned three SMG mutations with ICI-related OS benefits. The results demonstrated that *CFH* and *DGKG* mutations were markedly correlated with better OS outcomes in a specific sex (**Figure 2C**). Consistent with the aforementioned association of *CFH* mutations with ICI response, male patients with *CFH* mutations exhibited a significantly prolonged OS as compared with male patients without such mutations (log-rank test *P* = 0.042; **Figure 2D**). A similar result was observed in all patients (log-rank test *P* = 0.029; **Figure 2D**) but not in female patients. Interestingly, *DGKG* mutations were identified to associate with favorable survival outcomes in both male (log-rank test *P* = 0.003; **Figure 2D**) and female patients (log-rank test *P* = 0.016; **Figure 2D**), although the significant association of *DGKG* mutations with ICI response rate was not observed in female patients. Survival analysis showed that *PPP6C* mutations did not predict ICI outcomes in male, female, and overall patients (log-rank test all *P* > 0.05; **Figure S3**).

We also assessed the prognostic implications of the above three SMG mutations in melanoma patients from the TCGA cohort. The results showed that *CFH* and *DGKG* mutations were not correlated with the prognoses in any sex subgroups (log-rank test all *P* > 0.05; **Figure S4**). *PPP6C* mutations exhibited a significantly better survival outcome in male and overall patients (log-rank test *P* = 0.013 and 0.032, respectively; **Figure S4**) but not in female patients. Collectively, we determined several sex-dependent SMG mutations, which may be employed to evaluate the prognosis and immunotherapy efficacy in distinct sex subpopulations of melanoma.

### *CFH* Mutations Predictive of Better Immune Infiltration in Male Patients

**Figure 3:**
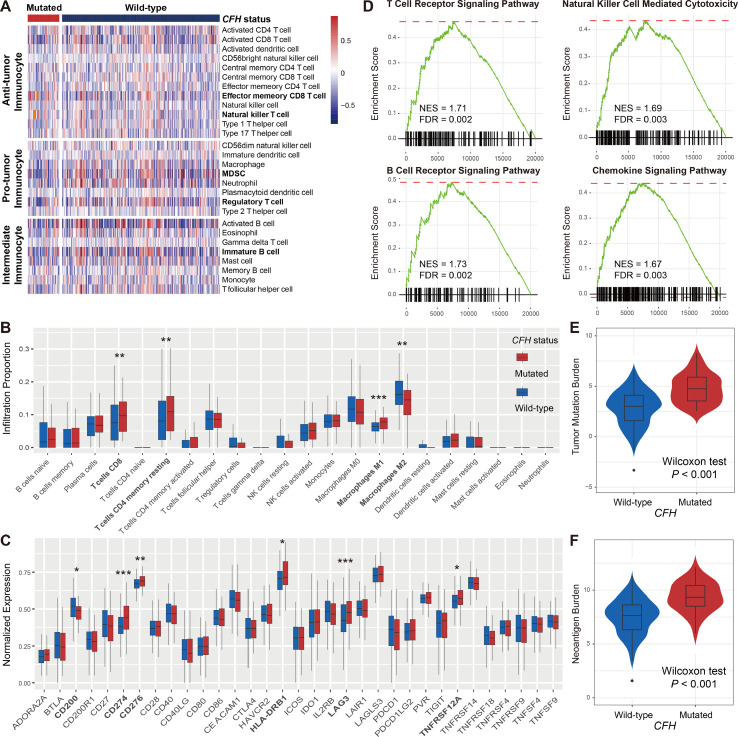
Lymphocyte infiltration, immune response pathways, and genomic features associated with *CFH* mutations in male patients. **(A)** Distinct immune cell subsets and **(B)** lymphocyte infiltration evaluation based on *CFH* mutational status achieved by the ssGSEA and CIBERSORT algorithm, respectively. **(C)** Boxplot representation of the expression of 33 immune checkpoint genes stratified by *CFH* status. **(D)** The immune response-related pathways of *CFH*-mutated patients obtained from the GSEA method. The varied distributions of **(E)** TMB and **(F)** NB in *CFH* mutated *versus* wild-type subgroups. Factors highlighted in bold were identified to be statistically different in distinct sex groups. *P < 0.05; **P < 0.01; ***P < 0.001.

Considering the vital roles of *CFH* mutations in predicting both response rate and survival benefits in male patients, we further investigated the potential immune-related mechanisms behind *CFH* mutations in the male subgroup. To ascertain the association of *CFH* mutations with immune cell infiltration, we created a heatmap using the ssGSEA approach to visualize the relative abundance of 28 infiltrating immune cell subtypes (**Figure 3A**). Antitumor immunocyte subsets, including effector memory CD8^+^ T cells and natural killer T cells, were significantly increased in male samples with *CFH* mutations, whereas protumor immunocyte subsets, such as MDSC and regulatory T cells, were markedly decreased in this subgroup (Wilcoxon rank-sum test all *P* < 0.05; **Figure 3A**). Additionally, we assessed the abundance of tumor-infiltrating lymphocytes in the melanoma tumor microenvironment using the gene expression data. We observed that CD8^+^ T cells, CD4^+^ memory-resting T cells, and M1 macrophages were more enriched in the *CFH*-mutated male subgroup, whereas M2 macrophages were less enriched in this subgroup (Wilcoxon rank-sum test all *P* < 0.05; **Figure 3B**).

In addition, comprehensive immune checkpoints were curated and analyzed. Results showed that expression of the well-known immune checkpoint, *CD274* (PD-L1), was significantly elevated in *CFH* mutant male patients. Other checkpoints, including *CD200*, *CD276*, *HLA-DRB1*, *LAG3*, and *TNFRSF12A*, were also observed to have higher expression in this group (Wilcoxon rank-sum test all *P* < 0.05; **Figure 3C**). The GSEA on gene expression profiles against the KEGG dataset demonstrated that enrichment of genes involved in T-cell receptor signaling, B-cell receptor signaling, natural killer cell-mediated cytotoxicity, and chemokine signaling pathway was significantly altered in the *CFH*-mutated male subgroup (all FDR < 0.01; **Figure 3D** and **Figure S5**).

Due to the important roles of TMB and NB in predicting immunotherapy efficacy, we compared the distinct distribution of TMB and NB between *CFH*-mutated and wild-type groups in male patients. Male patients with *CFH* mutations exhibited a markedly higher TMB than those without such mutations (median TMB: 4.75 *vs*. 3.01, Wilcoxon rank-sum test *P* < 0.001; **Figure 3E**). Similarly elevated NB was also observed in the *CFH*-mutated male group (median NB: 9.34 *vs*. 7.64, Wilcoxon rank-sum test *P* < 0.001; **Figure 3F**).

For comparison, we also evaluated lymphocyte infiltration and immune response pathways associated with *CFH* mutations in female patients. Although higher TMB and MB were enriched in the female patients with *CFH* mutations (**Figure S6A**), no significant lymphocyte infiltration (e.g., CD8^+^ T cells) and immune response-related pathways were observed in this subgroup (**Figures S6B, C**). Overall, *CFH* mutations in male patients may be a novel indicator for evaluating immune infiltration and ICI efficacy.

### Sex-Dependent Mutational Signatures Associated With ICI Efficacy

**Figure 4:**
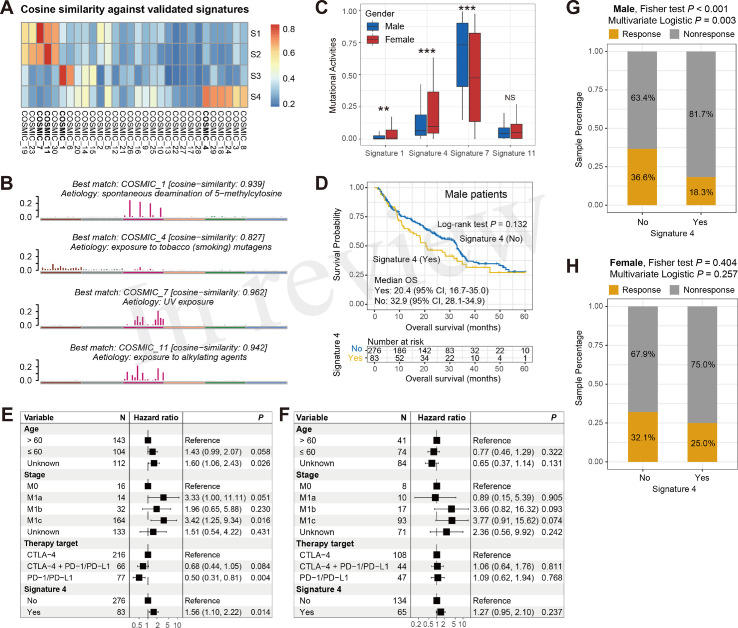
Determination of melanoma mutational signatures and their sex biases with respect to ICI efficacy. **(A)** Heatmap representation of the cosine similarities between the extracted four mutational signatures and 30 COSMIC signatures. **(B)** Identification of the four mutational signatures with specific etiology according to the COSMIC nomenclature. **(C)** Distinct activities of the identified four mutational signatures between male and female subgroups. **(D)** Kaplan–Meier survival curves stratified by signature 4 status in male melanoma patients. Forest plot illustration of the associations of signature 4 with ICI overall survival in **(E)** male and **(F)** female patients. Bar plots representation of the associations of signature 4 with ICI response rate in **(G)** male and **(H)** female patients. **P < 0.01; ***P < 0.001; NS, not significant.

The overall mutational pattern of the integrated melanoma cohort featured C > T (or G > A) mutations with a mutational proportion of 86.7% (**Figure 1A**). We extracted a total of four mutational signatures (i.e., S1–S4) using the Bayesian NMF method from this cohort and subsequently compared them with the 30 curated mutational signatures from COSMIC. The cosine similarity between the abovementioned four signatures and the 30 COSMIC signatures was calculated and presented as a heatmap (**Figure 4A**). Finally, signatures 1, 4, 7, and 11 were identified according to the COSMIC nomenclature (**Figure 4B**). The distributions of the four mutational signature activities across all patients are shown in **Table S3**. The clock-like signature 1, characterized by C > T mutations at CpG dinucleotides, is associated with age-related accumulation of spontaneous deamination of 5-methylcytosine. The mutational profile of signature 4, which is characterized by C > A mutations, was demonstrated to associate with exposure to tobacco-based carcinogens (e.g., benzo[*a*]pyrene). Mutational profiles of signatures 7 and 11, which exhibited the main mutations of C > T and were predominantly detected in melanoma, are likely due to exposure to ultraviolet (UV) light and treatment with alkylating agents, respectively. The differential analysis showed that female patients harbored significantly higher activities of signatures 1 and 4 as compared with male patients, whereas enhanced signature 7 activities were enriched in male groups (Wilcoxon rank-sum test all *P* < 0.01; **Figure 4C**). No difference was observed in relation to signature 11 between the two subgroups (Wilcoxon rank-sum test *P* > 0.05; **Figure 4C**).

To investigate whether these four mutational signatures contribute to the ICI response or resistance and further determine their sex disparities in relation to ICI efficacy, we used survival analyses and Cox regression models to assess the correlations. In all patients, the presence of signature 4 was identified to be associated with a significantly worse OS (log-rank test *P* = 0.009, multivariate Cox *P* = 0.005; **Figure S7A**), and a markedly prolonged OS was observed in patients with signature 11 (log-rank test *P* = 0.033, multivariate Cox *P* = 0.018; **Figure S7B**). The associations of signatures 1 and 7 with ICI survival were not significant (log-rank test both *P* > 0.05, multivariate Cox both *P* > 0.05; **Figures S7C, D**).

We further evaluated the sex disparities in the association of signatures 4 and 11 with ICI efficacy. The male subgroup with signature 4 exhibited a marginally significantly worse OS as compared with the male group without this signature (median OS: 20.4 *vs*. 32.9 months, log-rank test *P* = 0.132; **Figure 4D**). This result was more significant when controlling for confounding variables (i.e., age, stage, and ICI therapy types) in the multivariate Cox regression model (HR: 1.56, 95% CI: 1.10–2.22, *P* = 0.014; **Figure 4E**). Nevertheless, the link between signature 4 and inferior survival outcome was not observed in the melanoma female subgroup (HR: 1.27, 95% CI: 0.95–2.10, *P* = 0.237; **Figure 4F**). Similarly, a significantly decreased ICI response rate was also found in male patients with signature 4 (18.3% *vs*. 36.6%, Fisher exact test *P* < 0.001, multivariate logistic *P* = 0.003; **Figure 4G**). Likewise, no significant difference in response rates was observed between the signature 4 subgroups in female patients (25.0% *vs*. 32.1%, Fisher exact test *P* = 0.404, multivariate logistic *P* = 0.257; **Figure 4H**). For signature 11, there were no significant associations between the presence of this signature and ICI survival benefit in both male and female subgroups (log-rank test both *P* > 0.05, multivariate Cox both *P* > 0.05; **Figures S8A–D**).

### Identification of an ICI Resistance Molecular Subtype in Female Patients

The extracted mutational activities of four signatures from the aggregated cohort gave us an opportunity to investigate potential molecular subtypes relevant to ICI survival benefits by using the clustering method. We conducted a consensus clustering analysis with cluster numbers selected from 2 to 10. A favorable clustering consensus was observed when clustering ranks were chosen as 4 or 6 (**Figure S9A**). More subtle subgroups could be virtually microdissected with an increase in clustering numbers as illustrated in the cluster tracking plot (**Figure S9B**). Therefore, we selected six clusters (i.e., C1–C6) to evaluate their associations with ICI survival outcomes in distinct sex groups.

**Figure 5:**
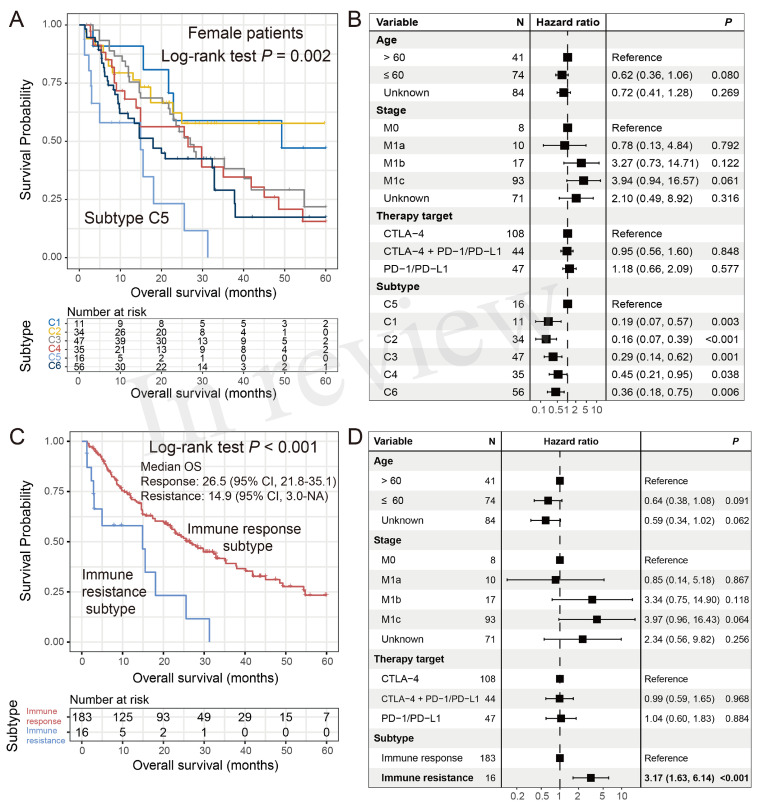
Identification of an ICI resistance subtype in female patients. **(A)** Kaplan–Meier survival curves stratified by the identified six clusters. **(B)** Forest representation of the association of the six clusters with ICI survival outcome. **(C)** Kaplan–Meier survival analysis and **(D)** multivariate Cox regression model of the defined “immune resistance” and “immune response” subtypes.

In the female subgroup, Kaplan–Meier survival analysis revealed significantly distinct OS outcomes, with patients from the C5 cluster [16 of 199 patients (8.1%)] exhibiting the poorest outcome as compared with the other five clusters (log-rank test *P* = 0.002; **Figure 5A**). In the multivariate Cox model with adjusted confounding factors, we treated the C5 cluster as the reference group and found that all five clusters exhibited better ICI prognoses (all HR < 1 and all *P* < 0.05; **Figure 5B**). In this study, we termed the female C5 cluster as the “immune resistance” subtype and the remaining five clusters were named as the “immune response” subtype. The significantly inferior ICI prognosis of the immune resistance subtype was demonstrated when comparing it with the immune response subtype in univariate survival analysis [median OS: 14.9 (95% CI, 3.0–NA) *vs*. 26.5 (95% CI, 21.8–35.1) months; log-rank test *P* < 0.001; **Figure 5C**] and multivariate Cox regression model with age, stage, and therapy types taken into account (HR: 3.17, 95% CI: 1.63–6.14, *P* < 0.001; **Figure 5D**). The findings obtained from this section reveal an immune molecular subtype that contributed to the failure of ICI treatment in female melanoma patients.

We also conducted correlation analyses behind the six clusters and ICI prognosis in male patients. Kaplan–Meier survival analysis showed that there were no significant differences between these six clusters, although one cluster exhibited the trend of the best survival (log-rank test *P* = 0.576; **Figure S10A**). We subsequently made a survival comparison between the group with the best survival and the integrative group containing the other five clusters. Likewise, no significant differences in ICI survival were observed in the survival analysis and multivariate Cox regression model (log-rank test *P* = 0.348, multivariate Cox *P* = 0.228; **Figure S10B**).

## Discussion

In this study, we conducted an integrated immunotherapy genomic analysis of 631 melanoma patients and found that mutations in several SMGs were associated with ICI efficacy in distinct sex subgroups. We revealed a male-dependent mutational signature (i.e., tobacco smoking-related signature) that was resistant to ICI treatment. In addition, we identified an immune molecular subtype as a poor prognosticator for female patients with melanoma.

Complement factor H (CFH), a complex innate immune surveillance system, is a fluid phase regulator of the complement protein. Recent studies have demonstrated the roles of CFH expression and its polymorphism in multiple chronic inflammatory diseases (41–44). In this immunotherapy research, *CFH* mutations exhibited the benefits of both the response rate and survival interval in male melanoma patients. Further mechanistic exploration revealed that the hot microenvironment represented by favorable lymphocyte infiltration and immune response pathways was markedly enriched in this subgroup. Nevertheless, a relatively cold immune microenvironment has been observed in *CFH*-mutated female patients. These findings indicate the potential of *CFH* mutations as a predictor of ICI efficacy in male melanoma patients.

PPP6C is a well-known driver gene in melanoma that regulates cell cycle progression (45). Although *PPP6C* mutations were associated with a higher response rate in male patients, no significant association was observed in relation to ICI survival. Interestingly, among the patients in the TCGA cohort, we found that male patients with *PPP6C* mutations harbored a significantly prolonged OS compared with those without such mutations. This suggests that in male patients, *PPP6C* mutations could predict survival outcomes in settings without immunotherapy.

Besides, we also investigated the connection between the identified three SMG mutations and ICI efficacy in melanoma samples with distinct treatment types. Response rate association analysis showed that only *DGKG* mutations were revealed to associate with a significantly elevated ICI response rate (52.9% *vs*. 26.4%) in patients who received anti-CTLA-4 agents; other associations did not reach statistical significance (**Figure S11A**). Survival analysis demonstrated that melanoma patients with *CFH* or *DGKG* mutations harbored significantly improved survival outcomes as compared with those wild-type patients in the group of anti-PD-l/PD-L1. In addition, *DGKG* mutations were also identified to associate with the prolonged anti-CTLA-4 treatment survival (**Figure S11B**). These findings suggest that immunotherapy efficacy predictive patterns of specific gene mutations were varied in melanoma patients with distinct treatment types, and further detailed and precise treatment strategies were needed.

Mutational signatures are unique combinations of mutation types generated by different mutational processes. In addition, sex differences in mutational signature activities have also been reported in several recent studies (10, 46). In this study, we revealed that age-related signature 1 and smoking-related signature 4 were markedly enriched in the female patients. However, an elevated activity of UV exposure-relevant signature 7 was observed in the male group. Noticeably, further sex-stratified analysis of the identified signatures demonstrated that smoking-related signature 4 was associated with inferior ICI survival and lower response rate in male patients, but not in females, indicating the sex dependence of signature 4 while evaluating ICI efficacy. Signature 4 in this aggregated cohort was identified to associate with the worse ICI outcome (**Figure S7A**). However, this smoking signature was predictive of a favorable outcome in ICI-treated lung tumors (47), suggesting the tumor heterogeneity of association of signature 4 with ICI efficacy. Therefore, immunotherapy strategies for the smoking-related signature should be determined based on the situations of distinct sex and cancer types.

The immune molecular subtypes were commonly detected based on the microenvironment features extracted from the transcriptomic data. Nevertheless, recent multiple immunotherapy studies have mainly focused on the somatic mutation level, and fewer studies have focused on gene expression profiles. In this aggregated melanoma research, only four of eight cohorts harbored the mRNA expression data, and it may be inappropriate to perform molecular subtyping using the expression data with the limited coverage of patients. The utilization of mutational signature activities extracted from melanoma samples is a good choice for determining the immanent subtype (21). Based on the activities of the four mutational signatures in this study, we identified six clusters and subsequently determined their associations with ICI survival in distinct sex subgroups. Noticeably, one cluster in female patients, named as the “immune resistance” subtype, exhibited the poorest OS outcome. However, no significant prognosis-related subtype was identified in the male group. Identification of the female-dependent immune subtype would provide clues for tailoring sex-based therapeutic regimens.

We integrated the genomic and clinicopathologic information of 631 melanoma patients and uncovered several sex-dependent determinants of ICI treatment. The heterogeneity of the included studies and the sample size of this assembled cohort remain major limitations. Overall, our study indicates clinical sex-related predictors and highlights the significance of future larger studies to robustly detect sex-related biomarkers predictive of immunotherapy outcomes.

## Data Availability Statement

The original contributions presented in the study are publicly available. This data can be found here: https://xenabrowser.net/datapages/?cohort=GDC%20TCGA%20Melanoma%20(SKCM)&removeHub=https%3A%2F%2Fxena.treehouse.gi.ucsc.edu%3A443.

## Ethics Statement

All studies have been approved by the Institutional Research Board.

## Author Contributions

QW and SW designed the study. QW, SW, FS, and WZ developed the methodology and acquired the related data. FS, WZ, QW, SW, YCY, YTY, JZ, MX, and CS performed the data analysis and interpretation. FS, WZ, QW, and SW drafted and revised the manuscript. QW and SW supervised the study. All authors contributed to the article and approved the submitted version.

## Funding

This study was supported by the National Natural Science Foundation of China (Nos. 81872719 and 81803337), Provincial Natural Science Foundation of Shandong Province (No. ZR201807090257), and National Bureau of Statistics Foundation Project (No. 2018LY79).

## Conflict of Interest

The authors declare that the research was conducted in the absence of any commercial or financial relationships that could be construed as a potential conflict of interest.

## Publisher’s Note

All claims expressed in this article are solely those of the authors and do not necessarily represent those of their affiliated organizations, or those of the publisher, the editors and the reviewers. Any product that may be evaluated in this article, or claim that may be made by its manufacturer, is not guaranteed or endorsed by the publisher.
