# Supplementary material for: Sex Disparities of Genomic Determinants in Response to Immune Checkpoint Inhibitors in Melanoma
Source: Front Immunol. 2021 Nov 2;12:721409. doi: 10.3389/fimmu.2021.721409 (PMC8594267; doi:10.3389/fimmu.2021.721409)
Supplement: Supplementary file 1 [file DataSheet_1.docx]

**Supplementary Figures**


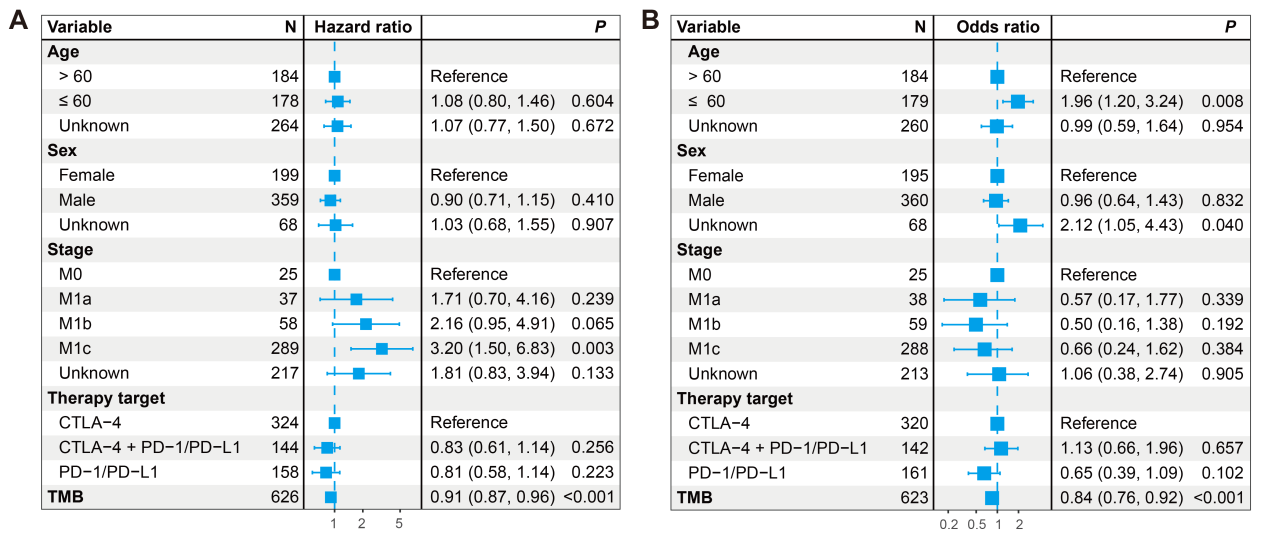


**Figure S1.** Forest representation of the associations of the TMB with (A) ICI overall survival outcome and (B) response rate in the multivariate regression models with age, sex, stage, and therapy types taken into account in the pooled cohort.


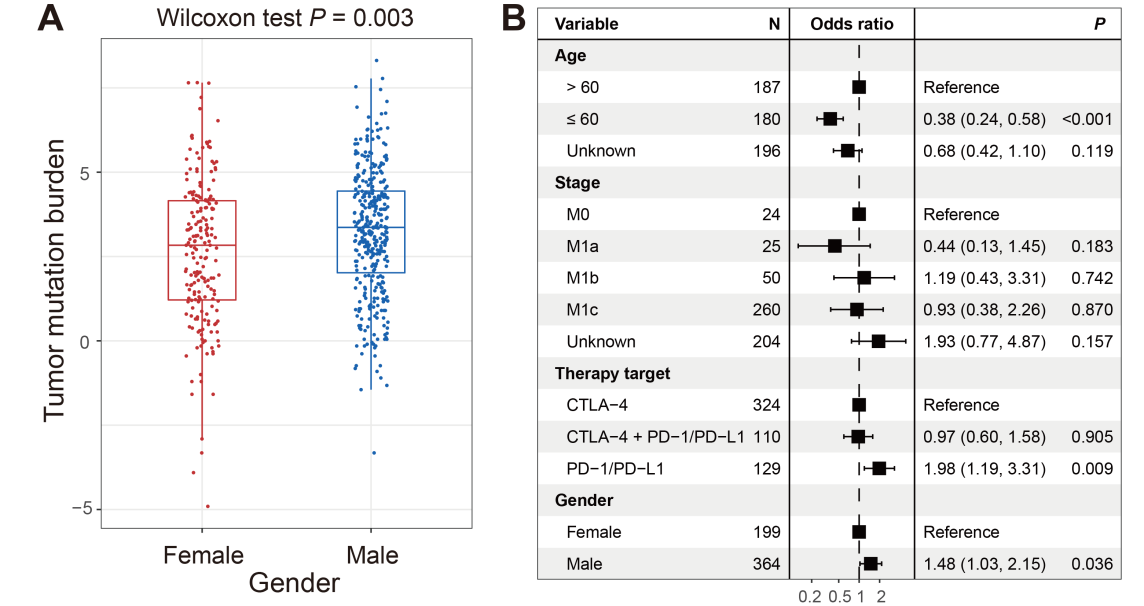
**Figure S2.** Distinct distribution of TMB in sex subgroups. (A) Box plot representation of the association of distinct sex with the TMB. (B) Multivariate Logistic regression model illustration of the association of male and female subgroups with TMB.


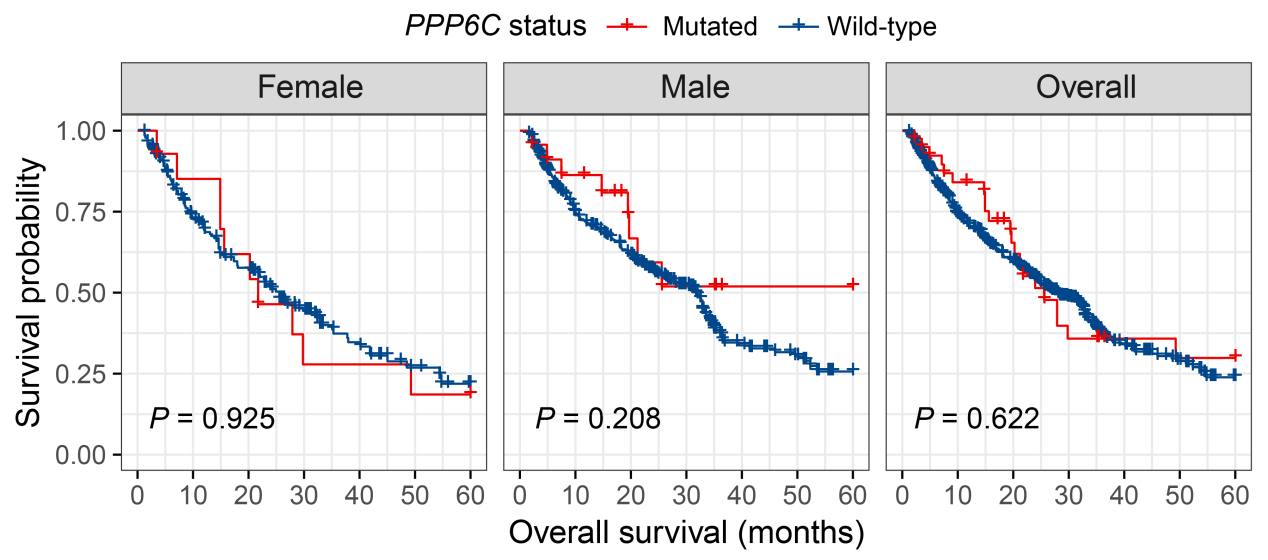


**Figure S3.** The ICI prognostic implications of the *PPP6C* mutations in male, female and overall groups of the integrated melanoma cohort.


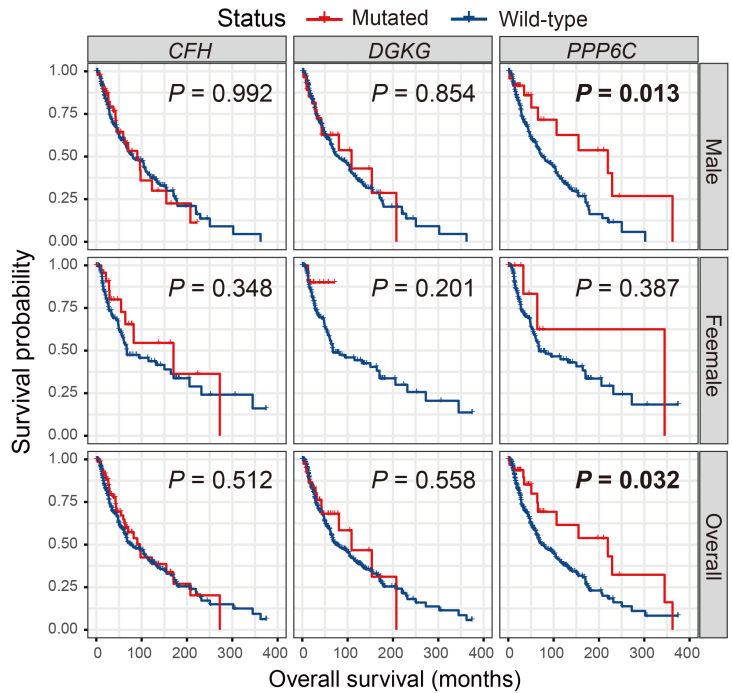


**Figure S4.** Association of *CFH*, *DGKG*, and *PPP6C* mutations with survival outcomes in distinct sex subgroups in the TCGA cohort.


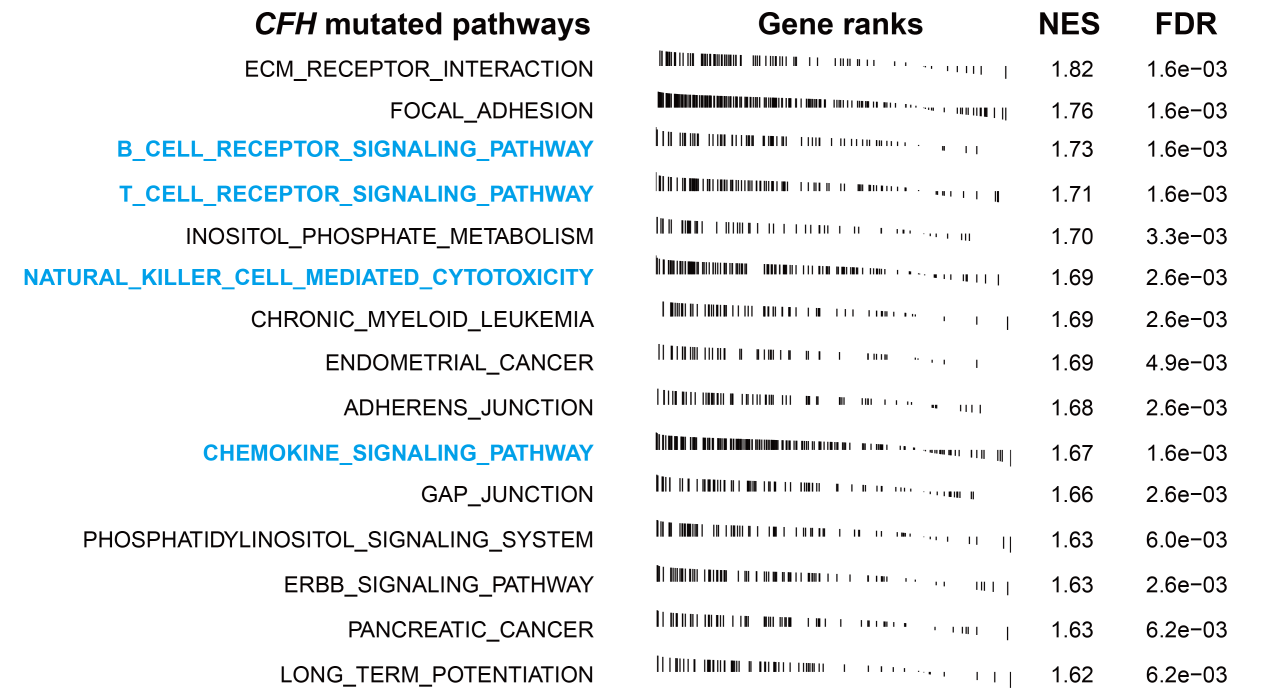


**Figure S5.** The top significantly enriched pathways in the subgroup of *CFH*-mutated male melanoma patients. Pathways highlighted with blue indicate the immune response relevant pathways.


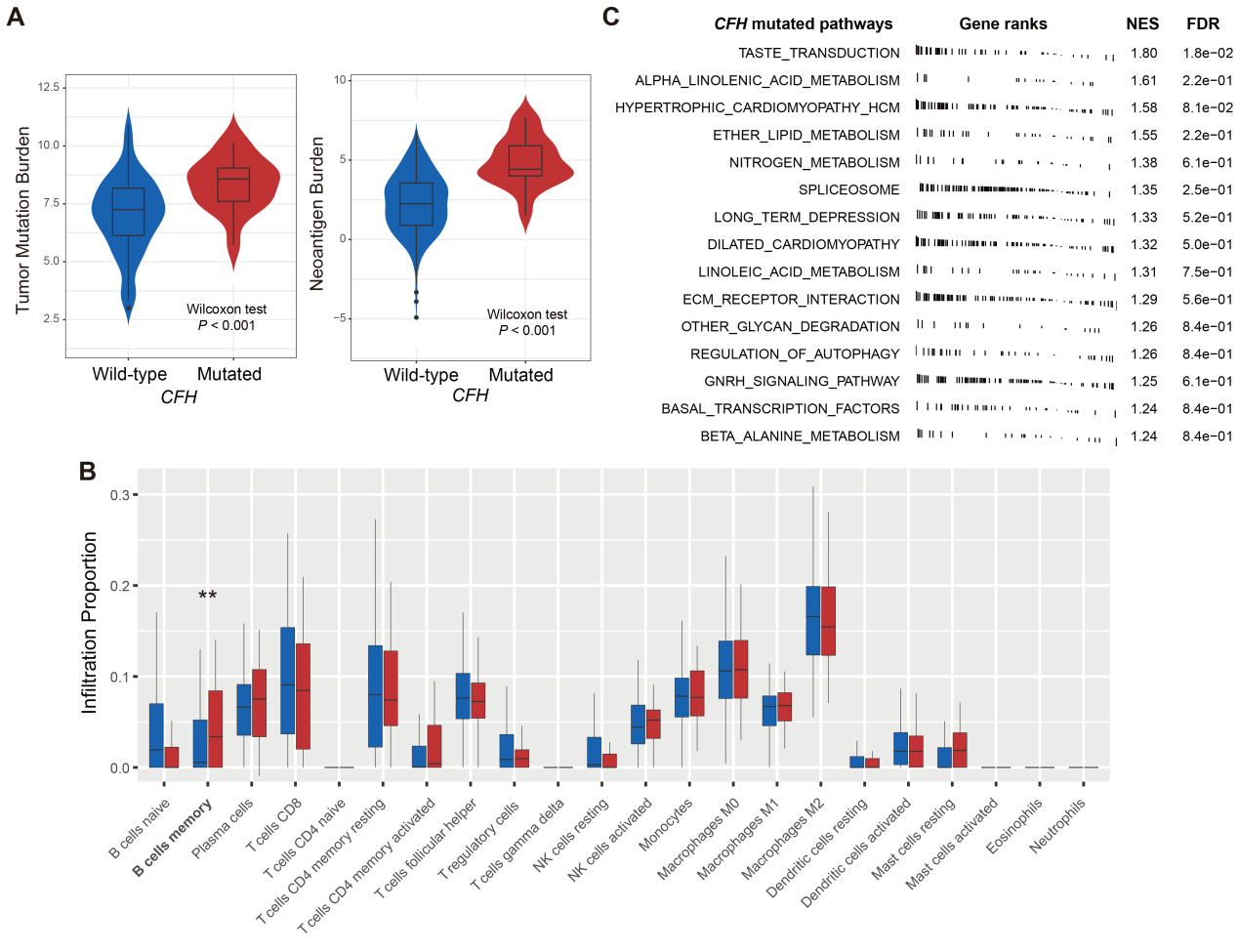


**Figure S6.** Associations of *CFH* mutations with (A) mutational burden, (B) lymphocytes cells infiltration, and (C) enriched pathways in female melanoma patients. ** *P* < 0.01

**
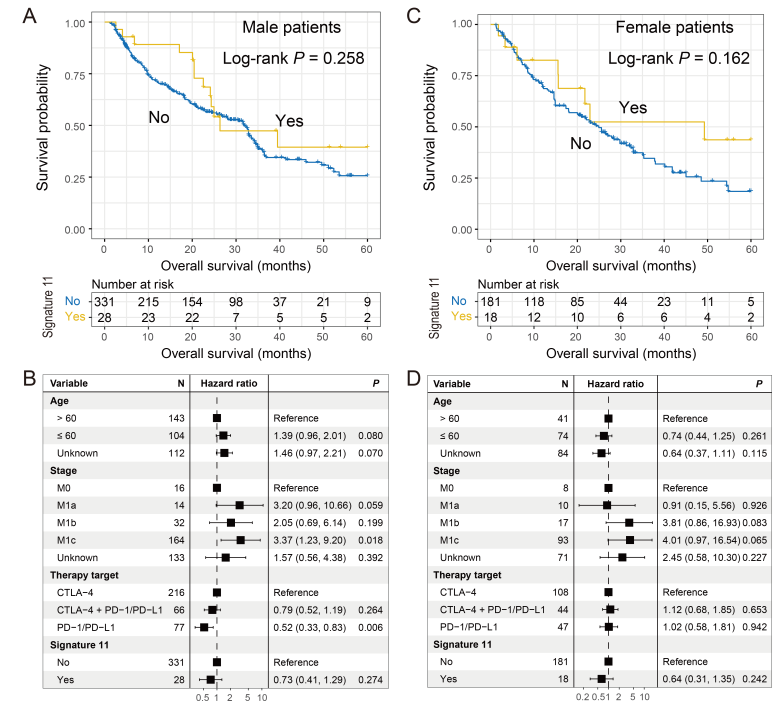

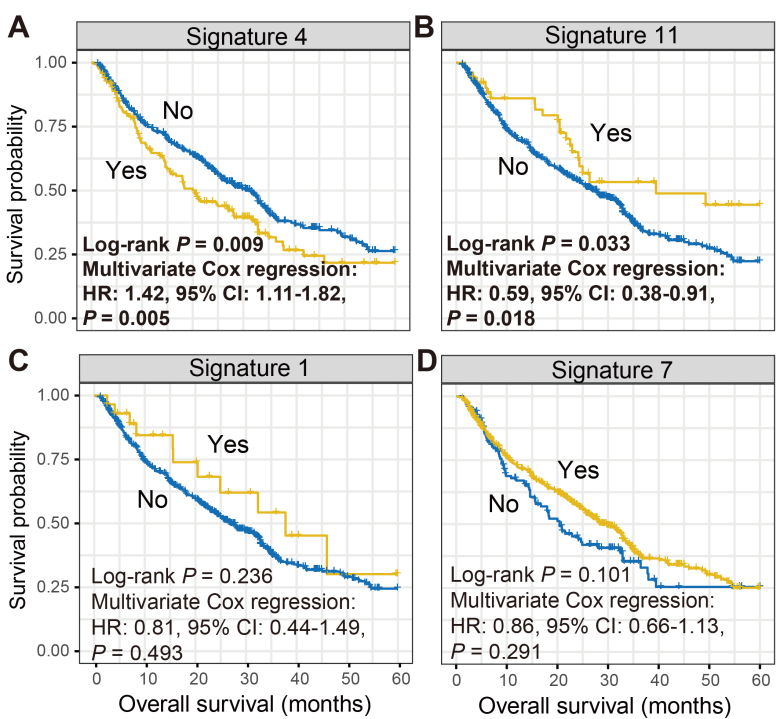
Figure S7.** Kaplan-Meier survival curves stratified by the status of (A) signature 4, (B) signature 11, (C) signature 1, and (D) signature 7 in overall ICI-treated melanoma patients.

**Figure S8.** Association of the presence of signature 11 with ICI survival in distinct sex groups. (A) Kaplan-Meier survival analysis and (B) multivariate Cox regression model representation of the association of signature 11 with ICI prognosis in male patients. (C) Kaplan-Meier survival analysis and (D) multivariate Cox regression model representation of the association of signature 11 with ICI survival in female patients.

**
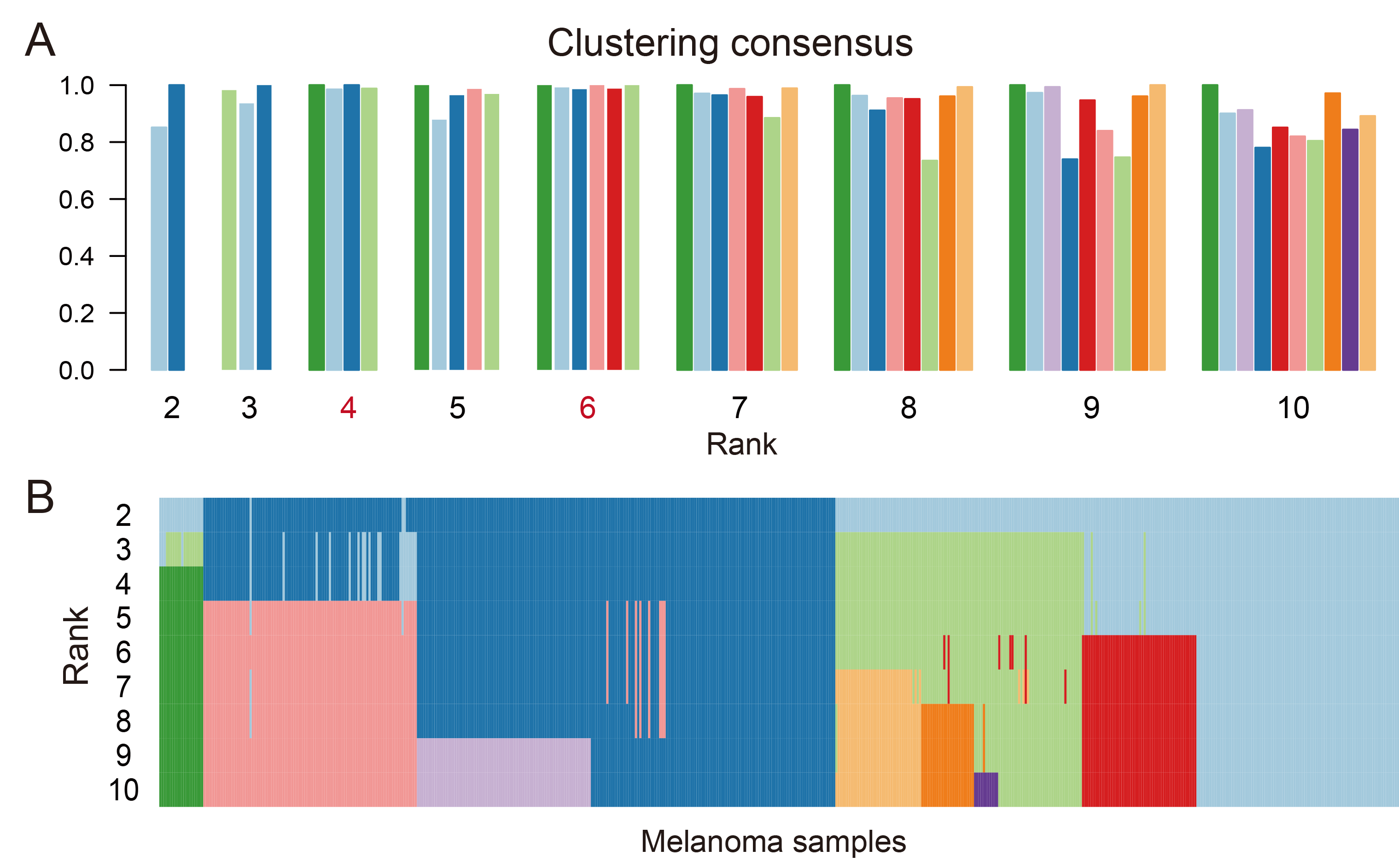
Figure S9.** Clustering performance under the condition of distinct clustering ranks. (A) Clustering consensus exhibition based on the ranks from 2 to 10. The values colored with red indicate better clustering ranks. (B) The clustering tracking plot with the rank varying from 2 to 10.


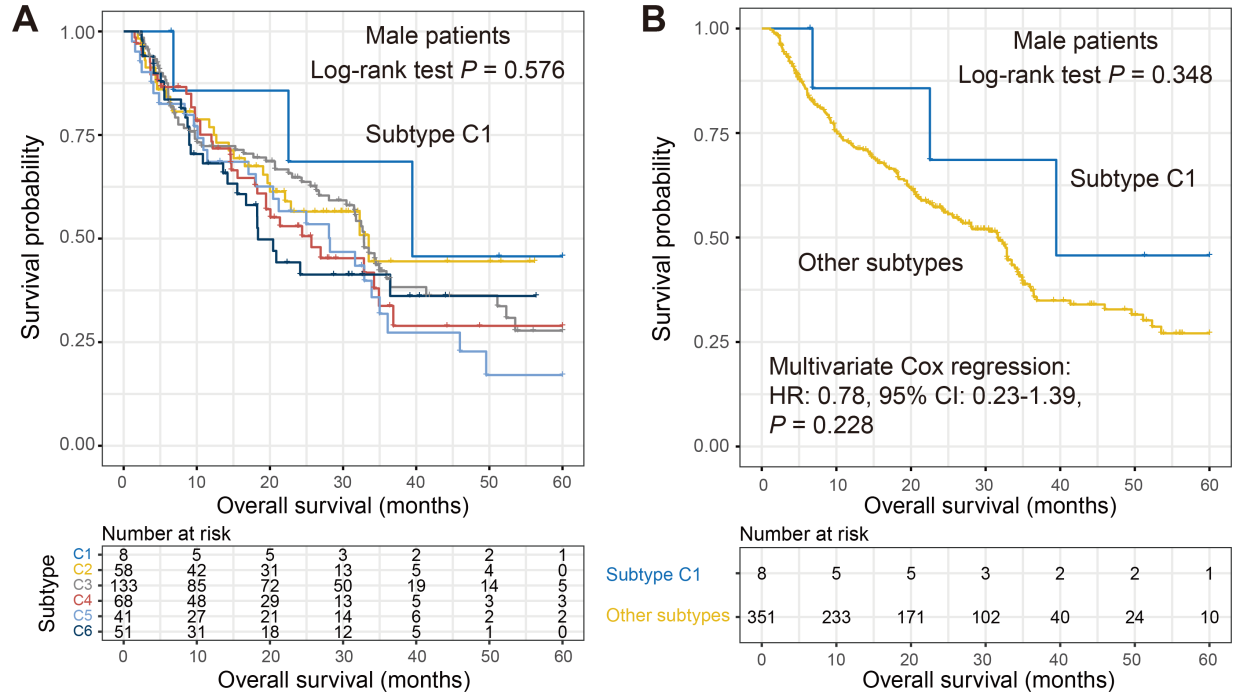


**Figure S10.** Distinct Kaplan-Meier survival curves stratified by (A) the identified 6 clusters and (B) different groups in melanoma male subgroup.


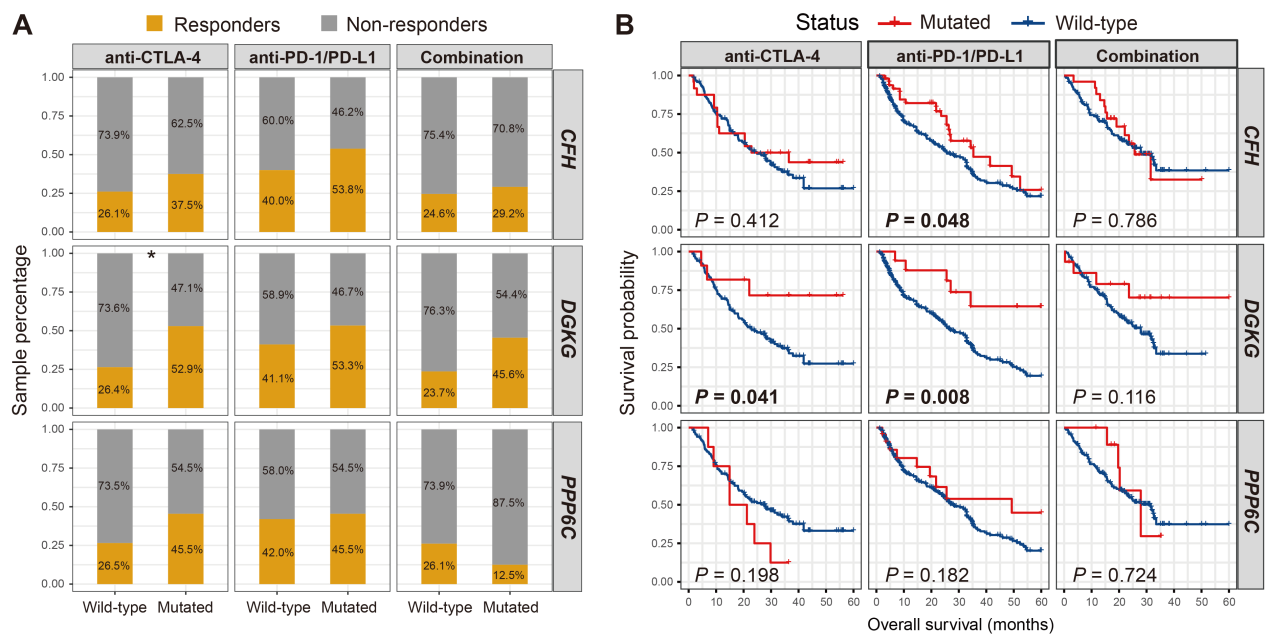


**Figure S11.** The associations of identified 3 SMG mutations with immunotherapy (A) response rate and (B) survival outcome in melanoma patients treated with distinct ICI types.
